# Long-Acting Gel Formulations: Advancing Drug Delivery across Diverse Therapeutic Areas

**DOI:** 10.3390/ph17040493

**Published:** 2024-04-12

**Authors:** Hossein Omidian, Renae L. Wilson

**Affiliations:** Barry and Judy Silverman College of Pharmacy, Nova Southeastern University, Fort Lauderdale, FL 33328, USA; rw1273@mynsu.nova.edu

**Keywords:** hydrogel therapies, long-acting gels, chronic diseases, localized treatment, prolonged release

## Abstract

This multifaceted landscape of long-acting gels in diverse medical fields, aims to enhance therapeutic outcomes through localized treatment and controlled drug release. The objective involves advancements spanning cancer treatment, immunotherapy, diabetes management, neuroendocrine disorders, ophthalmic applications, contraception, HIV/AIDS treatment, chronic diseases, wound care, and antimicrobial treatments. It explores the potential of long-acting gels to offer sustained and extended drug release, targeted therapy, and innovative administration routes while addressing limitations such as scalability challenges and regulatory hurdles. Future directions focus on personalized therapies, biodegradability, combination therapies, interdisciplinary innovation, regulatory considerations, and patient-centric development. This comprehensive review highlights the pivotal role of long-acting gels in transforming therapeutic approaches and improving patient outcomes across various medical conditions.

## 1. Introduction

The ongoing research and development of long-acting gels across different medical disciplines are driven by the necessity to improve drug delivery systems and therapeutic outcomes. For cancer treatment, the creation of long-acting gels is vital for enhancing the efficacy of treatments and reducing adverse effects. Notably, there is a concerted effort to amplify the anti-cancer properties of drugs such as tamoxifen and to craft controlled release mechanisms that offer localized and prolonged therapy for cancer patients [1,2,3,4,5,6,7].

The field of immunotherapy and postoperative care is witnessing the development of long-acting gels to extend the duration of immunogenic chemotherapy, boost the immune response, and diminish systemic toxicity. This indicates a growing need for treatments that provide both sustained release and targeted action [8,9,10]. In diabetes management, long-acting gels are crucial for the continuous and controlled delivery of medications to effectively regulate blood glucose levels, reflecting a trend towards enhancing patient convenience and treatment success [11,12,13,14,15,16,17].

Research in neuroendocrine disorders and acromegaly has demonstrated a shift towards long-acting formulations, such as lanreotide Autogel, to ensure consistent and manageable treatment options for patients, with the goal of optimizing therapeutic effectiveness and improving quality of life [18,19,20,21,22,23,24,25,26,27,28,29]. The investigation into ophthalmic applications and pain management reveals a significant demand for long-acting gels to improve adherence to treatment, reduce the frequency of administration, and increase patient comfort in managing conditions like glaucoma and chronic pain [30,31,32,33,34,35,36,37,38,39,40,41].

In the area of contraception and reproductive health, there is a push for the development of long-acting gels to provide dependable, long-term solutions for fertility control and urogenital health, highlighting the necessity of treatments that are both effective and user-friendly [42,43,44,45,46]. The HIV/AIDS treatment and prevention sector is progressively adopting long-acting formulations to guarantee consistent drug delivery, thereby enhancing the practicality and efficacy of antiretroviral therapies [47,48,49,50].

Research on chronic diseases and conditions, including Parkinson’s disease, schizophrenia, and neonatal hyperinsulinism, has led to the creation of long-acting gels to enable better disease management and enhance patients’ quality of life [51,52,53]. In wound care and antimicrobial treatments, the emphasis on long-acting formulations [54,55] underlines the essential need for prolonged antimicrobial activity and therapeutic effects in wound management.

The progress in enhanced drug delivery technologies and targeted therapies aims to improve the bioavailability, stability, and patient compliance of treatments for various conditions, propelling the development of long-acting gels to meet these complex therapeutic needs [56,57,58,59,60,61,62,63].

**Scheme 1:**
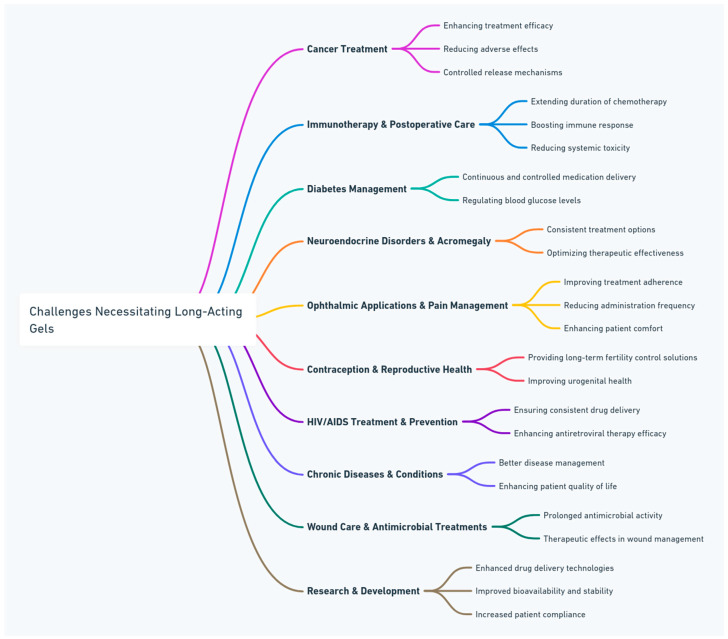
Long-acting gels in medicine necessity and impact across diverse applications.

Scheme 1 visualizes the various challenges and needs across multiple medical disciplines that necessitate the use of long-acting gels. It highlights specific areas like cancer treatment, immunotherapy, diabetes management, neuroendocrine disorders, pain management, reproductive health, HIV/AIDS treatment, chronic diseases, and wound care. For each area, it outlines key factors such as enhancing treatment efficacy, improving patient convenience, and extending drug release. This organized representation aids in understanding how long-acting gels contribute to advancing medical treatments and improving patient outcomes.

## 2. Long-Acting Gel Compositions

Long-acting gel compositions are being extensively researched for their potential in delivering medication directly to the target site within the body. These gels are primarily characterized by their temperature-sensitive properties, where they transition from a liquid to a gel state upon reaching body temperature. Notable materials in this category include N-isopropylacrylamide (NIPAm)-based gels, which solidify at body temperature [3], and Poloxamer 407, a thermosensitive polymer that forms a gel at physiological temperatures [8,37,38,61].

Another significant development in gel technology is the creation of pH-sensitive and redox-responsive gels. These are engineered to release their drug payload in response to specific environmental triggers, such as changes in the pH level or the redox state within the body. Examples include redox-active injectable gels [10] and hydrogels that modulate drug release according to pH fluctuations [39].

Chemical crosslinking techniques are employed to stabilize gel structures, utilizing agents or processes that create strong, stable networks within the gel material. For instance, gelatin–hydrazide and aldehyde-functionalized polyethylene glycol (PEG) form crosslinked hydrogels [2]. In contrast, physical crosslinking methods rely on interactions that are not covalent, leading to gels that are often reversible and environmentally responsive. Collagen and hyaluronic acid gels [6] exemplify this, forming networks through temperature or pH-induced changes, while Tetra-PEG hydrogel microspheres [27] represent another approach to physical crosslinking [17].

The use of polymeric networks is prevalent in the formulation of long-acting gels. These networks, created from both synthetic and natural polymers, are designed for specific drug delivery applications. Chitosan-based gels [5,46,47,64] are particularly noted for their biocompatibility and gel formation through ionic crosslinking. Similarly, poly(lactic-co-glycolic acid) (PLGA)-based gels [7,13,40,42,43,60] are valued for their biodegradability and capacity for sustained drug release.

The incorporation of nanostructures and composite materials into gels has opened new avenues for enhanced functionality and efficiency. This includes the use of nanoparticle-loaded gels, such as those containing curcumin and doxorubicin nanoparticles within a poloxamer matrix [8], and composite gels like dammar gum-ethyl cellulose microsponge formulations [65].

In situ-forming hydrogels represent a frontier in gel technology, designed to form gels directly at the application site, thereby improving drug delivery efficiency and targeting. These gels can be triggered by various stimuli, including temperature and pH changes, or by interacting with bodily fluids. Noteworthy examples include hydrogel systems based on collagen and hyaluronic acid [6], as well as formulations combining Metoprolol tartrate with Carbopol and Pluronic for in situ gelation [30].

The advancement of long-acting gel compositions is marked by innovations in material science and engineering, allowing for the development of gels that are not only responsive to bodily conditions, but also capable of providing controlled and targeted drug delivery.

## 3. Long-Acting Gels for Advanced Drug Delivery

The development of long-acting hydrogels innovations stems from a convergence of disciplines including materials science, nanotechnology, and pharmacology, each contributing to the creation of more effective, safe, and patient-friendly treatments. The strategic focus on hydrogel composition is essential to overcoming the limitations of conventional therapies, aiming to provide sustained drug release, improved stability, and enhanced bioadhesion, thereby improving patient outcomes and adherence to treatment.

Cancer treatment, diabetes management, neuroendocrine disorders, ophthalmology, pain management, contraception, sexual health, HIV prevention, and other disease states have revealed a shared goal: to develop delivery systems that are not only efficient and versatile, but are also tailored to meet the needs of specific patient populations. This is achieved through the use of biodegradable polymers, bioadhesive properties, and innovative formulation strategies, including the integration of nanotechnology and hybrid material systems.

### 3.1. Long-Acting Gels in Cancer Treatment

**Figure 1:**
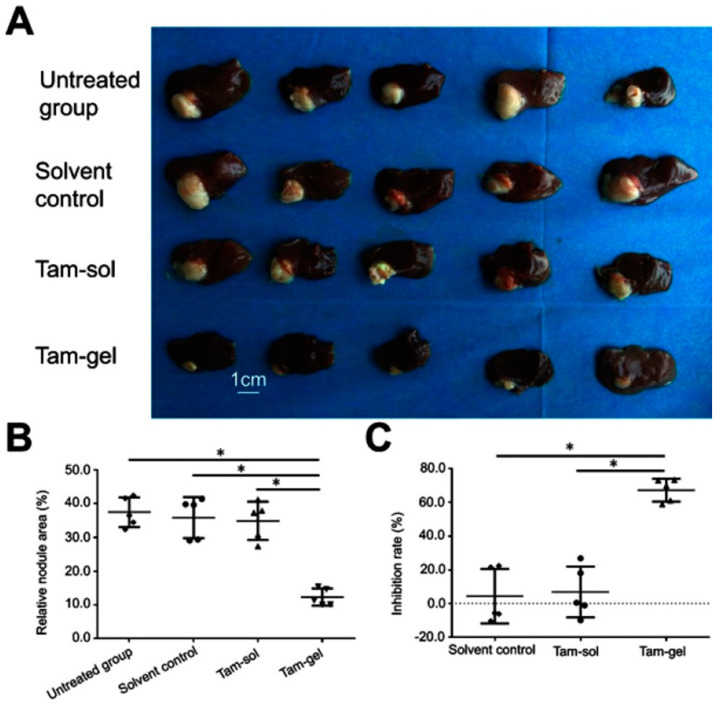
Shows results after a one-dose administration of Tam-Gel and its ability as a long-acting agent to inhibit intrahepatic subcutaneous tumor growth of MCF-7 cells in nude mice (**A**) Photographs of intrahepatic lesions caused by MCF-7 in immunodeficient rats. (**B**) Quantitative results of the lesions’ area by total liver area (mean ± SD). (**C**) Inhibition rates calculated from the quantitative results. * *p* < 0.05 [1].

Recent advancements for cancer treatment have focused on developing hydrogels for targeted and sustained drug delivery. A temperature-sensitive phase-change hydrogel named Tam-Gel, designed for tamoxifen delivery, was tested for its slow-release capabilities and antitumor effects using mouse and rat models. In both subcutaneous and intrahepatic breast cancer models, Tam-Gel demonstrated an enhanced local treatment potential and a reduction in side effects compared to traditional treatment methods, showing promise for improving cancer therapy (Figure 1) [1].

Another significant development involved an injectable, self-healing, pH-responsive gelatin-PEG/Laponite hybrid hydrogel loaded with doxorubicin (DOX). This hybrid hydrogel was evaluated for its gelation, injectability, biocompatibility, and drug release profile, along with its effect on cancer cell lines. The results indicated its potential for localized, controlled drug delivery, minimizing systemic toxicity and improving treatment outcomes [2].

Researchers also created an injectable, near-infrared/pH-responsive nanocomposite hydrogel loaded with DOX, aimed at extended release and enhanced photothermal therapy. This hydrogel was tested for its mechanical properties, swelling behavior, drug release, and photothermal efficiency, focusing on its application in chemophotothermal synergistic therapy. The findings suggested that this hydrogel could lead to better tumor targeting and reduced side effects, owing to its localized treatment and sustained drug presence [3].

A temperature–ion–pH-responsive hydrogel made from glutathione–gellan gum conjugate loaded with DOX was synthesized and tested for optimized drug release and anticancer activity. This hydrogel demonstrated selective tumor targeting, a reduced impact on healthy cells, and effective anticancer activity, indicating its potential for improving cancer treatment modalities [4].

A chitosan-based thermoreversible injectable hydrogel loaded with PEGylated melphalan was developed, with evaluations conducted on its release profile and stability. The assessments of its gelation time, rheological properties, and drug release behavior pointed towards an improved therapeutic profile for melphalan, suggesting advancements in localized cancer therapy with decreased systemic toxicity and less frequent dosing [5].

Furthermore, a NanoCD hydrogel combining curcumin and doxorubicin was created and examined for its potential in enhancing postsurgical cancer treatment. The hydrogel’s effects on reactive oxygen species generation and immunogenic cell death were studied, with in vivo tests assessing local chemotherapy and immune response activation in post-resection tumor models. This research suggested that the NanoCD@Gel could offer an effective postsurgical cancer treatment strategy by boosting antitumor immunity and reducing tumor recurrence [8].

Continuing with advancements in cancer treatment, a hydrogel incorporating gold nanorods (AuNRs) and macrophage migration inhibitory factor (MIF) inhibitors was developed for combined photothermal and immune therapy. Through in vitro and in vivo studies, the hydrogel’s impact on cancer cell proliferation, migration, and immune cell infiltration was analyzed, indicating its potential as a long-acting immunotherapy that could decrease cancer recurrence and boost the immune response [9].

A redox-active, polyamine-poly(ethylene glycol)-polyamine triblock copolymer, poly(acrylic acid), protein in redox-active injectable gel (RIG) utilizing a polyion complex for sustained protein delivery was designed and its mechanical strength and protein release profile were examined. Subcutaneous injections of the protein-loaded RIG in tumor-bearing mice were tested, revealing its capability to inhibit tumors and minimize systemic toxicity, suggesting a promising local sustained-release protein therapy system [10].

A composite dammar gum–ethyl polymeric microsponge-based gel (D-MSPG) for the controlled release of mupirocin was formulated, with evaluations of its morphology, size, and drug release profile. The gel’s physical properties were characterized, and ex vivo skin penetration studies were conducted and compared to marketed formulations. This approach may provide an improved topical therapy strategy with controlled drug delivery and enhanced treatment effectiveness [65].

Researchers also developed a bioinspired in situ gelling curcumin-loaded nanoparticle/hydrogel composite using collagen and hyaluronic acid, tailored for in situ gelling in ocular therapy. The composite’s biocompatibility, degradation, and drug release were assessed in vitro, along with its antiproliferative effects on human uveal melanoma cells. This development holds potential for the long-term treatment of uveal melanoma, offering a reduced injection frequency and improved patient compliance [6].

An innovative approach involved loading both free and nanoparticle-encapsulated BCNU into a natural extracellular matrix (ECM) hydrogel, tested for sustained drug release and tumor inhibition in a rat glioblastoma model. The hydrogel was injected into post-resection tumor cavities in rats, with tumor growth inhibition monitored over 30 days, showing promise for improving post-surgical outcomes in glioblastoma by providing sustained local chemotherapy to prevent recurrence [7].

Furthermore, Rv-Soy protein granules were developed, encapsulated in sodium alginate films to create Rv nanocomposite in situ gelling films. These were assessed for their drug release, encapsulation efficiency, and anticancer activity, with the optimized formulation’s cytotoxicity, apoptotic activity, and gene expression impact tested on colorectal cancer cells. This method suggests a promising approach for colorectal cancer treatment with controlled release and enhanced cellular response [66]. Table 1 shows the objectives, gel compositions, methods of administration, and corresponding references for various studies aimed at cancer treatment and drug delivery systems.

### 3.2. Long-Acting Gels in Diabetes Management

**Figure 2:**
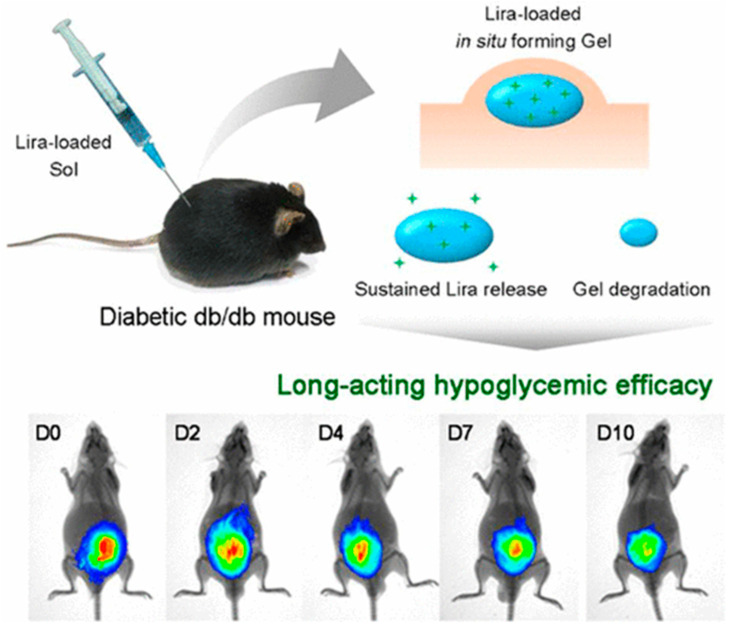
Injectable thermosensitive PEG–polyester hydrogel formulation of liraglutide as a long-acting treatment for type 2 diabetes mellitus. D0, D2, D4, D7 and D10 represent the long-acting hypoglycemic effect [11].

In the field of diabetes treatment, significant advancements have been made through the development of hydrogel systems for sustained drug delivery. Researchers developed a poly(epsilon-caprolactone-co-glycolic acid)-poly(ethylene glycol)-poly(epsilon-caprolactone-co-glycolic acid) (PCGA-PEG-PCGA) thermosensitive PEG-polyester hydrogel for the continuous release of liraglutide, which was analyzed for its duration of action and drug release characteristics in vivo. The hydrogel demonstrated effectiveness in lowering blood sugar levels in diabetic mice, suggesting its potential as a once-weekly antidiabetic treatment that could enhance glycemic control and patient adherence (Figure 2) [11].

**Figure 3:**
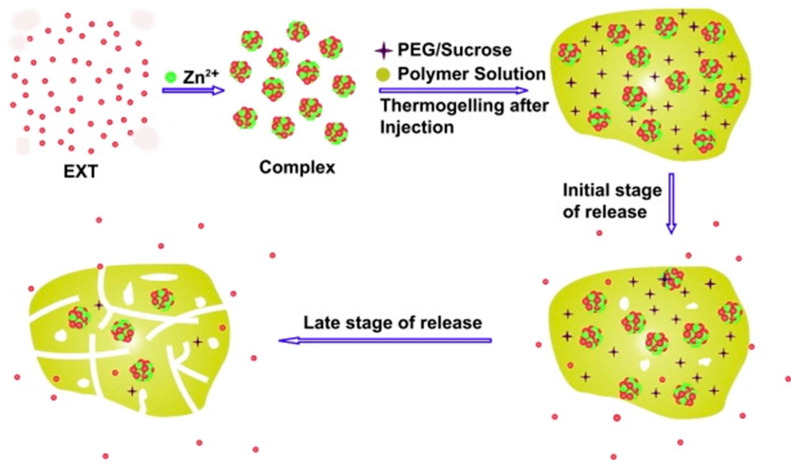
A schematic presentation of the drug encapsulation and delayed drug release from optimized zinc-EXT nanoparticles encapsulated into the physical polymeric hydrogel [12].

Another study utilized a thermoreversible PLGA-PEG-PLGA hydrogel for the delivery of exenatide, with the release rate modifiable by zinc acetate and other additives. The hydrogel was tested in vivo for its ability to regulate blood glucose, showing a sustained drug effect over a week. This research indicates the feasibility of a weekly diabetes treatment regimen that could offer stable glucose control and decrease the need for frequent injections (Figure 3) [12].

A novel approach was taken in creating mixed hydrogels for a Depot-gel-in-Ms-in-Matrix system, which encapsulated exenatide within microspheres and a PLGA-PEG-PLGA and PCLA-PEG-PCLA hydrogel matrix. This system was evaluated over 46 days for its in vitro release and in vivo efficacy, successfully maintaining stable blood glucose levels and body weight in diabetic mice, pointing to its promise as a long-acting diabetes treatment that preserves drug bioactivity and enhances glucose regulation [13].

An injectable phospholipid gel, comprising phospholipid S100, medium-chain triglycerides (MCT), and ethanol, was developed for the stable delivery of exenatide. The pharmacokinetics and therapeutic efficacy of this gel were tested in diabetic animal models, showing potential for extended blood glucose control in type II diabetes with less frequent injections [14].

Researchers also produced decanoic acid-modified glycol chitosan hydrogels that contained palmitic acid-modified exendin-4, exploring their release characteristics and therapeutic potential. The hypoglycemic effect and sustained release of exendin-4 were assessed in diabetic mice, suggesting that this could be an effective long-term treatment for type 2 diabetes with the benefit of reducing the frequency of dosing [15].

Inhalable nanogels carrying palmityl-acylated exendin-4 were formed using deoxycholic acid-modified glycol chitosan. These were tested for lung deposition and their ability to lower blood sugar. The prolonged hypoglycemic effect of the inhaled nanogels in diabetic mice, along with an assessment of lung tissue, indicated the potential for a long-acting inhaled antidiabetic treatment with extended action [16].

Hydrogel microparticles were developed through physical and chemical crosslinking for the oral delivery of insulin. This was aimed at examining insulin loading, release, and bioavailability. The hypoglycemic effect and insulin bioavailability were tested in a type 2 diabetes mouse model, showcasing the potential to improve diabetes management through oral delivery, thus reducing the reliance on injections and enhancing patient compliance [17]. Table 2 shows the objectives, gel compositions, methods of administration, and corresponding references for various studies focused on developing long-acting antidiabetic systems and formulations for diabetes treatment.

### 3.3. Neuroendocrine and Other Tumor Treatments

In a series of clinical evaluations, the efficacy of lanreotide Autogel (LAR) was thoroughly assessed in patients with metastatic well-differentiated neuroendocrine tumors (NETs). These studies administered 120 mg of lanreotide Autogel monthly to patients, closely monitoring their symptom relief, changes in tumor markers, and disease progression. The findings indicate that lanreotide Autogel not only provides effective relief from symptoms, but also controls tumor growth in patients with well-differentiated metastatic NETs, exhibiting a favorable safety and tolerability profile [18]. Additionally, a retrospective analysis over 24 months supported these results, confirming lanreotide Autogel’s role in managing symptoms and limiting tumor progression with minimal side effects [19].

Further research pooled data from multiple trials involving lanreotide depot dosages ranging from 60 to 120 mg in patients with acromegaly, particularly focusing on those new to treatment. These multicenter trials evaluated the drug’s ability to regulate hormone levels in both treatment-naive patients and those post-surgery, demonstrating its effectiveness in achieving biochemical control across different patient histories [20]. Another study compared the effects of lanreotide Autogel with previous treatments in managing acromegaly, revealing that the switch to lanreotide Autogel maintained its efficacy and even improved tolerability over the long term [21].

A comprehensive four-year follow-up on acromegaly patients who had undergone surgery revealed that long-term treatment with lanreotide Autogel consistently controlled growth hormone (GH) and insulin-like growth factor I (IGF-I) levels. However, it highlighted the need for the ongoing monitoring of glucose metabolism to ensure patient safety and treatment effectiveness [22]. Furthermore, a phase III clinical trial evaluated lanreotide Autogel in treatment-naive acromegaly patients over a period of 48–52 weeks. This study confirmed the high efficacy and safety of the treatment, noting significant improvements in symptoms and the overall quality of life of the patients [23].

Further studies compared lanreotide Autogel with octreotide LAR in acromegalic patients, focusing on dosing intervals adjusted based on GH levels to monitor hormonal control and its clinical effectiveness. An open, multicenter longitudinal study transitioned patients from octreotide LAR to lanreotide Autogel, with dosing adjustments driven by effectiveness. The outcomes illustrated the efficacy of lanreotide Autogel and suggested its potential for less frequent dosing in the management of acromegaly [24]. Additionally, research evaluated insulin resistance and beta-cell function in acromegalic patients treated with lanreotide Autogel against those untreated, utilizing HOMA indices. This prospective, cross-sectional study highlighted lanreotide Autogel’s impact on glucose metabolism, pointing towards the need for customized diabetes management in treated patients [25].

A crossover study further examined the comparative effectiveness of lanreotide Autogel and octreotide LAR in controlling GH and IGF-I levels in acromegalic patients. This study, allowing patients to switch between the two treatments, confirmed the efficacy of both, providing flexibility in the therapeutic approach to acromegaly with different somatostatin analogues [26]. Research extended into the development of Tetra-PEG hydrogel–octreotide conjugates with a self-cleaving linker, testing for slow release and extended serum half-life in rats. This preclinical study of the hydrogel–octreotide conjugate aimed at sustained drug release demonstrated potential for an improved and less painful administration method, with prolonged activity suitable for treating acromegaly and NETs [27].

A simulated-use study assessed the preferences of nurses between the new lanreotide Autogel syringe and the octreotide LAR syringe, considering various attributes. Conducted across multiple nations, this study evaluated the experiences and preferences of healthcare providers, indicating that lanreotide Autogel might offer an enhanced experience for healthcare providers and subsequently better patient care [28]. Lastly, a 12-month randomized crossover study on acromegaly patients compared the effects of lanreotide (LAN) and octreotide (OCT) on GH and IGF-I levels. Patients received treatment with either LAN or OCT for six months each, with regular hormonal and clinical evaluations. The study suggested that switching between LAN and OCT could benefit certain patients, especially those experiencing treatment failure or adverse effects [29]. Table 3 presents the objectives, gel compositions, methods of administration, and corresponding references for various studies investigating the efficacy and safety of lanreotide autogel in treating neuroendocrine tumors and acromegaly.

### 3.4. Ophthalmology and Vision Health

Innovative approaches to ophthalmology and vision health regarding delivery drugs are enhancing the treatment of various eye conditions. Researchers formulated metoprolol tartrate in Carbopol 934 and Pluronic F127 gels, testing their pH balance, in vitro drug release, rheological behavior, and effects on intraocular pressure (IOP) in rabbits. The evaluation covered aspects like clarity, pH stability, rheological characteristics, compatibility, and drug release efficiency, with IOP effects measured in rabbits. The findings indicate that metoprolol tartrate gel formulations could provide the extended control of intraocular pressure, offering a significant improvement in glaucoma treatment [30].

Similarly, atenolol was incorporated into carboxymethylcellulose and sodium alginate gels, with studies focused on in vitro release and the prolonged drug effect on IOP in rabbits. These studies assessed the potential of atenolol gel as a long-acting ophthalmic formulation that could enhance the management of glaucoma [31].

The effect of beta-blocker gels on ocular aberrations was examined by applying a carteolol long-acting solution and timolol gel to healthy volunteers. Ocular wavefront aberrations were measured at various intervals after application, comparing the two beta-blocker formulations. This research highlighted the importance of considering the impacts of beta-blocker gels on the optical quality of the eye in the treatment of ocular conditions [32].

Innovative drug delivery systems such as injectable hydrogel rods designed for the sustained delivery of bevacizumab to the retina were also explored. These were compared with in situ-forming hydrogels and rod injectors through in vitro and in vivo studies, evaluating their drug release, stability, and anti-angiogenic effects over four months in animal models. Hydrogel rods emerged as a promising method for sustained drug delivery in retinal diseases, potentially reducing the frequency of treatments [33].

The encapsulation of BAY224 in biodegradable silica microparticles within a silica hydrogel was investigated for its controlled release both in vitro and in rabbits over 55 days. This method showed promise for long-acting intravitreal therapy, which could decrease the need for frequent injections and improve patient compliance in treating ocular diseases [34].

A study on bimatoprost-loaded nanovesicular in situ gelling implants for subconjunctival delivery evaluated their extended release and IOP-lowering effect in rats over two months with a single injection. This indicated the potential for these implants to serve as a long-acting treatment for glaucoma, reducing the dependence on daily eye drops [35].

A mucoadhesive thermogel composed of gelatin, poly(N-isopropylacrylamide), and lectin was developed for sustained drug release. Tested in a rabbit model of dry eye disease (DED), it showed long-lasting efficacy in repairing the corneal epithelium over 14 days. This suggests that the thermogel could be an effective, long-acting topical treatment for DED, improving drug bioavailability and patient comfort [36]. Table 4 outlines the objectives, gel compositions, methods of administration, and corresponding references for studies aimed at developing long-acting ophthalmic gels for extended drug contact and slow release, as well as other intravitreal and subconjunctival delivery systems for ocular diseases.

### 3.5. Pain Management and Anesthesia

Recent developments in pain management and anesthesia have focused on formulating gels and hydrogels for sustained analgesic delivery. A notable innovation is a thermosensitive, bioadhesive gel combining lidocaine and dexamethasone, optimized for intraperitoneal administration. This gel was specifically tailored in terms of gelation temperature and viscosity, and it was characterized in vitro to ensure sustained analgesic release following abdominal surgery. This approach may present a new strategy for managing postoperative pain, particularly in abdominal surgeries, by offering prolonged analgesia [37].

Researchers have also developed thermogels containing poloxamer and levobupivacaine aimed at providing sustained analgesic release. In vitro studies assessed the gel formulation for its drug release, permeation, and ease of syringe use. This could introduce a novel method for sustained pain management, potentially reducing the frequency of dosing and enhancing postoperative pain relief [38].

Another advancement is a biodegradable copolymer hydrogel incorporating bupivacaine microcrystals and calcium carbonate for pH-regulated sustained release. This hydrogel was evaluated for its in vitro dissolution and in vivo analgesic effect in rats, providing extended pain relief for up to 44h. This suggests the potential for prolonged postoperative pain management with less need for repeated medication [39].

**Figure 4:**
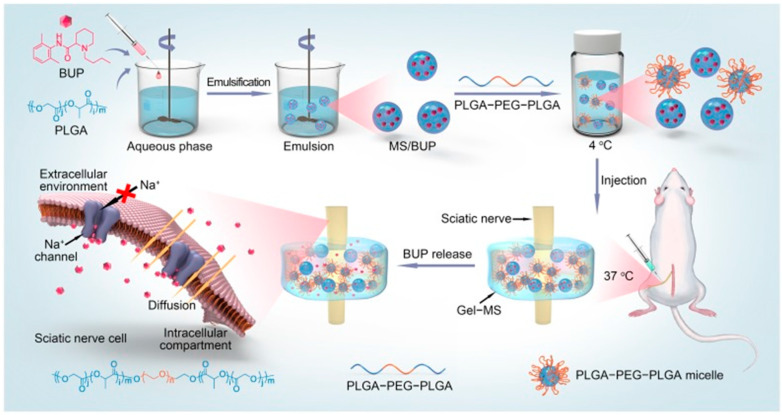
A schematic representation of the preparation and in vivo nerve blockade effect of the gel-–microsphere system with bupivacaine (Gel-MS/BUP) [40].

A gel–microsphere system containing bupivacaine was developed for controlled release, with analyses conducted on its in vitro drug release and in vivo analgesic effects. Its sustained pain relief and biocompatibility were assessed using a rat sciatic nerve block model, indicating a promising method for long-term pain relief, and potentially decreasing the frequency of analgesic administration (Figure 4) [40].

A composite of hydrogel and microspheres containing bupivacaine and dexmedetomidine was formulated for sequential drug release. Both in vitro and in vivo studies indicated extended analgesic effects with good biodegradability and compatibility, representing a potential approach for long-lasting, synergistic pain relief in clinical settings [41].

For patients with chronic wounds, a 4% lidocaine gel in a TRI-726 matrix was evaluated for its prolonged analgesic effect. A week-long study involving patients with various chronic wounds showed that a single application could significantly reduce pain levels, potentially decreasing the reliance on systemic pain medications and lowering the risk of medication abuse [67].

The pharmacokinetics of butorphanol in poloxamer 407 gel were investigated in Hispaniolan Amazon parrots. After subcutaneous administration, the analysis aimed to determine the duration of the analgesic effect, suggesting a novel method for managing pain in avian species with a single administration, which could streamline and improve pain management practices in veterinary care [68]. Table 5 summarizes the objectives, gel compositions, methods of administration, and corresponding references for studies focusing on the development of long-acting gels for postoperative pain management in abdominal surgeries, as well as other formulations for sustained analgesia and chronic wound pain management.

### 3.6. Contraception, Sexual Health, and HIV Prevention

Contraception, sexual health, and HIV prevention have led to the development of various long-acting delivery systems for use as therapeutic agents. A thermogel containing levonorgestrel was developed for sustained delivery in animals, utilizing a PLGA-PEG-PLGA copolymer to facilitate reversible sol–gel transition and controlled drug release. In vitro and in vivo studies in rats demonstrated the prolonged release of levonorgestrel, indicating its potential as an effective, long-acting contraceptive solution for livestock and pets, thereby reducing the administration frequency [42].

Levonorgestrel was also tested in a biodegradable gel matrix for subcutaneous delivery in cotton-top tamarins, with the gel composition and drug concentration optimized for contraceptive efficacy. This approach showed an extended contraceptive effect with minimal side effects, offering a viable solution for population management in endangered species and supporting controlled breeding programs [43].

A cross-sectional study assessed the use of long-acting reversible contraceptives (LARCs) and the factors influencing their utilization, employing interviews and logistic regression for data analysis. This study identified predictors of LARC use, underlining the need for enhanced family planning services and guiding strategies to improve contraceptive uptake [44].

Research into oxybutynin bioadhesive vaginal gels for overactive bladder (OAB) treatment compared the pharmacokinetics of these gels when administered orally to rabbits. The hydroxypropyl methylcellulose (HPMC)-based gel showed superior bioavailability and mucoadhesion, suggesting a potential long-acting treatment alternative for OAB and vaginal dryness that could improve patient compliance [46].

Focus groups were held with users of contraceptive intravaginal rings (IVRs) and lubricants to explore their sensory perceptions and the meanings they derived from product use. This research, grounded in perceptibility science and cultural theory, shed light on how product characteristics impact user experience and preferences, guiding the development of more acceptable vaginal health products [69].

Additionally, focus groups with women discussed their perceptions of long-acting vaginal gels for HIV prevention, evaluating gel prototypes to gain insights into acceptability and design preferences for anti-HIV microbicides. This suggests that long-acting vaginal gels could be favored for HIV/sexually transmitted infection (STI) prevention, emphasizing the importance of user-friendly product designs [70].

HIV treatment with the use of tenofovir alafenamide-chitosan nanoparticles in oleogels were created for extended anti-HIV drug release. This formulation was assessed for in vitro and ex vivo release, showing promise for chronic HIV treatment with the potential for less frequent dosing, thereby enhancing patients’ adherence and quality of life [47].

**Figure 5:**
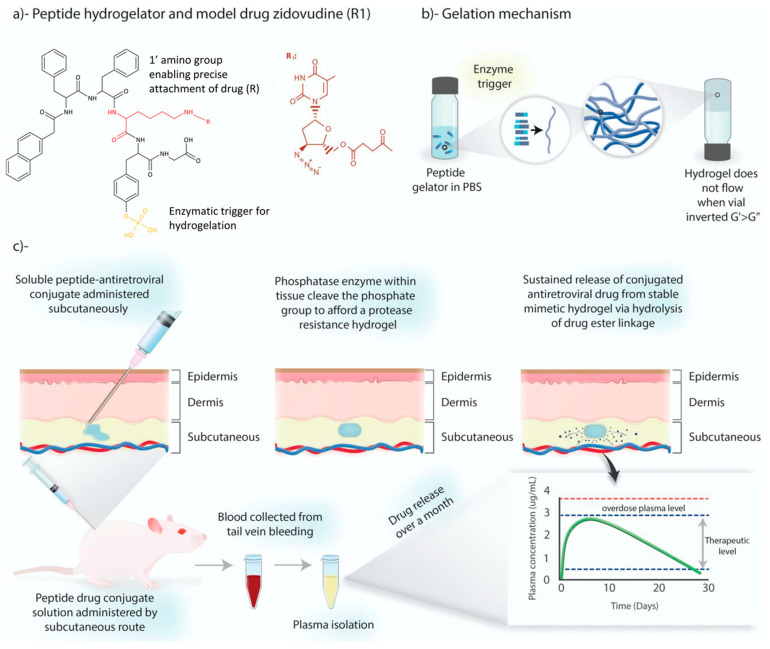
Peptide hydrogelators as an enzyme-triggered, long-acting injectable delivery platform for HIV/AIDS. (**a**) Chemical structure of L-*α* peptide hydrogelator NapFFKY(p)G-OH and covalently conjugated antiretroviral drug (R1). (**b**) Peptide gelators, upon exposure to an enzyme, lead to the formation of a 3D network of entangled fibers that entrap water and form a hydrogel. (**c**) Demonstrates the potential application of a soluble peptide–antiretroviral conjugate administered monthly via subcutaneous injection [48].

An injectable peptide hydrogel for the extended systemic delivery of zidovudine, an antiretroviral drug, was developed; this demonstrated in situ gel formation and sustained drug release in rat models, indicating its potential for long-term HIV management and the possibility of reducing treatment frequency (Figure 5) [48].

A qualitative study with stakeholders on long-acting injectable (LAI) antiretroviral therapy (ART) for HIV identified multi-level factors affecting the adoption of LAI ART, providing insights that could inform implementation strategies in Los Angeles County and improve HIV treatment uptake and adherence [49].

**Figure 6:**
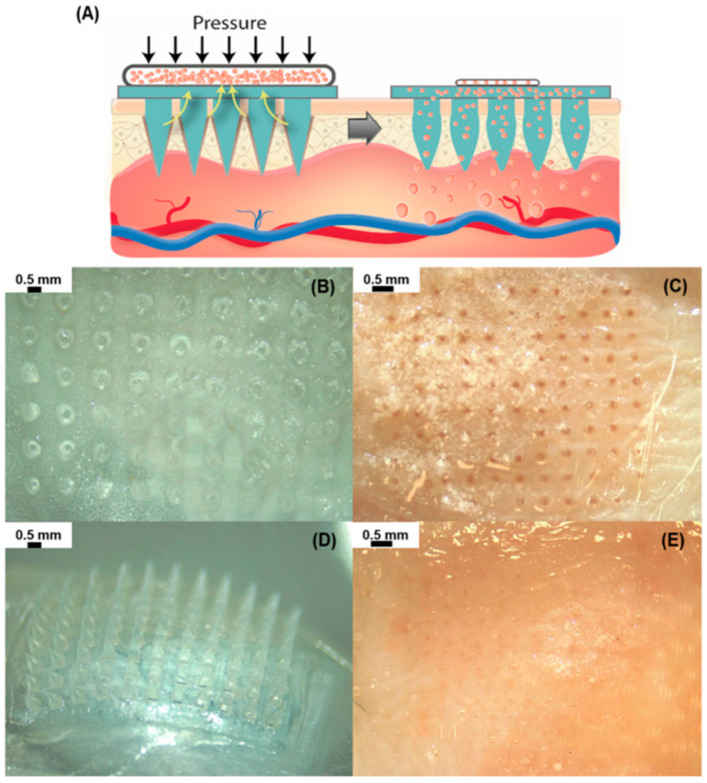
(**A**) Schematic representation of HF-MAP-mediated delivery from a reservoir. Digital light microscopy images with X12.5 magnification of (**B**) MAP1 after skin deposition experiment. (**C**) Skin surface where MAP1 was applied during skin deposition experiment, (**D**) MAP2 after skin deposition experiment (X8 magnification), and (**E**) skin surface where MAP2 was applied during skin deposition experiment [50].

Hydrogel-forming microneedle arrays (HF-MAPs) with cyclodextrin–drug reservoirs were developed for the sustained delivery of cabotegravir, and analyzed in ex vivo and in vivo settings for intradermal drug deposition and pharmacokinetics. The study evaluated cabotegravir sodium (CAB-Na) delivery in porcine skin and rats, demonstrating extended release and high drug deposition, heralding a new method for long-acting HIV prevention that could decrease the administration frequency (Figure 6) [50]. 

Table 6 provides an overview of the objectives, gel compositions, methods of administration, and corresponding references for studies aimed at developing long-term injectable contraception for animals, effective contraceptive options for specific species, and long-acting bioadhesive vaginal gels for various applications.

### 3.7. Other Therapeutic Areas

In the treatment of severe congenital hyperinsulinism (CHI) in infants, lanreotide Autogel has been used as a long-term therapy, with a study demonstrating improved blood glucose control and decreased rates of hypoglycemia. Lanreotide Autogel presents a less invasive, effective alternative for treating severe CHI in infants, potentially circumventing the need for surgery [51].

For Parkinson’s disease management, a sustained-release in situ gel of rasagiline was developed, optimized with aluminum hydroxide to ensure uniform release. Pharmacokinetic studies in rats indicated sustained rasagiline release, maintaining steady blood concentrations for one month, suggesting an improved treatment approach that could enhance medication adherence and clinical outcomes [52].

In schizophrenia treatment, an exposure–response analysis of RBP-7000, a long-acting risperidone formulation, was conducted. A Phase 3 study correlated plasma exposure with clinical outcomes, showing sustained efficacy and highlighting the influence of metabolic rates. This suggests the potential of once-a-month injections to improve schizophrenia management, enhancing patient adherence and outcomes [53].

A comparison of the effects of transdermal testosterone (T) gel and intramuscular testosterone undecanoate (TU) on the hematopoiesis and testosterone levels in hypogonadal men indicated that TU is more effective in increasing hematocrit and improving anemia, suggesting that long-acting TU may be more beneficial for certain hypogonadal men, especially in treating anemia [71].

A gel comprising Ginkgo biloba extract and a sodium alginate nanocomplex was developed and characterized for its wound-healing properties, including its size, encapsulation, rheology, and performance both in vitro and in vivo. The gel promoted wound healing through antioxidant activity, collagen production, and growth factor upregulation, demonstrating its potential for clinical wound care applications [54].

In the development of antimicrobial wound dressings, electrospun polycaprolactone (PCL)/gelatin fibers containing various concentrations of quaternary ammonium salts (QASs) were analyzed for their antimicrobial efficacy and material properties. These micro/nanofiber membranes exhibited broad-spectrum antibacterial activity, suitable mechanical properties, and minimal cytotoxicity, suggesting their potential as effective wound dressings to reduce infection risks in clinical settings [55].

Bioadhesive eutectogels containing drug nanocrystals were developed for mucosal tissue delivery, characterized by their rheological, elastic, and adhesive properties, along with in vitro drug release. The eutectogels exhibited extended drug release, superior mucosal adhesion, and enhanced drug deposition in mucosal tissues, indicating a novel approach for long-term mucosal drug delivery to improve treatment outcomes [72].

A chitosan-based nanogel for the in-situ generation of silver nanoparticles and the slow release of Ag^+^ ions was evaluated for its antibacterial and biofilm ablation activities. This nanogel showed a prolonged antibacterial effect and was effective in eradicating biofilms in implant infection models, demonstrating good biocompatibility and suggesting a promising treatment for implant-related infections with sustained antibacterial action [64].

Moreover, a cellulose nanocrystal aggregated gel containing donepezil was constructed for sustained release at physiological pH, with assessments of gelation, injectability, viscoelasticity, and drug release kinetics. In vivo studies showed that this gel extended the drug’s half-life and offered sustained release compared to standard formulations, indicating a novel method for prolonged drug delivery and potentially enhancing the treatment efficacy for conditions like Alzheimer’s disease [56].

Continuing with the innovative developments in drug delivery systems, an alginate in situ-forming injectable gel containing paliperidone palmitate was designed. The gelation time and drug release rates were modulated by varying the ratios of glucono-d-lactone and pyridoxal 5‘-phosphate, optimizing the gel strength and minimizing discomfort upon injection. In vitro studies demonstrated controlled drug delivery over four weeks, signifying a potential long-acting injectable system that could enhance patient compliance and treatment efficacy [57].

Silk fibroin xerogels were prepared using ethanol to promote gelation and cross-linking and were loaded with estradiol for extended drug delivery. In vitro analyses confirmed sustained estradiol release for up to 129 days, showcasing the potential for prolonged drug delivery systems [58].

A nifedipine-loaded gelatin microcapsule was developed, spray-dried, and then coated with Eudragit resin to achieve sustained release. Comparative studies in rats indicated that the coated microcapsules provided controlled release and the higher bioavailability of nifedipine compared to uncoated forms, suggesting an improved approach for long-acting oral delivery and enhanced patient compliance [59].

Research into the management of chronic rhinosinusitis involved a thermoresponsive hydrogel with mometasone furoate-loaded PLGA microspheres for extended drug release in the paranasal sinuses. Tested in a rabbit model, the hydrogel effectively reduced sinonasal inflammation over four weeks, offering a promising post-operative solution for prolonged steroid delivery in sinus treatment, potentially reducing the recurrence of chronic rhinosinusitis (CRS) [60].

The pharmacokinetics of moxifloxacin delivered via different routes, including intravenously, subcutaneously, and via a long-acting poloxamer 407 gel, were studied in rabbits. The long-acting formulation exhibited an extended half-life and suitable bioavailability, suggesting its effectiveness and tolerability for treating bacterial infections in rabbits [61].

A carboxymethylcellulose–agar hydrogel for the controlled release of chlorine dioxide was developed, with the release process based on a modified kinetic model following Fick’s diffusion law. This analysis indicated a diffusion-controlled release mechanism, extending the effective release time to two months, with potential applications in sustained disinfection across various industries [62].

Furthermore, hydrogel-forming microarray patches with solid dispersion reservoirs were developed for the transdermal delivery of hydrophobic atorvastatin. The system was tested in vitro, ex vivo, and in vivo, showing sustained transdermal delivery over 14 days in rats, indicating the potential for long-acting microdepot formation within the skin. This innovative system could offer a new method for the long-acting transdermal delivery of hydrophobic drugs, potentially improving patient compliance and therapeutic outcomes [63]. Table 7 summarizes the objectives, gel compositions, methods of administration, and corresponding references for various studies aimed at developing long-acting formulations for a range of medical applications, including treatments for neonatal hyperinsulinism, Parkinson’s disease, schizophrenia, wound healing, antimicrobial wound dressings, drug delivery systems for chronic sinusitis, and the transdermal delivery of hydrophobic drugs, among others.

## 4. Methods of Administrations of Long-Acting Gels

The administration of long-acting gels in drug delivery systems is finely tuned to the specific needs of each treatment, ensuring that medications are delivered effectively and with minimal discomfort to the patient. For targeted cancer therapy, tamoxifen’s hydrogel formulation (Tam-Gel) is designed for direct injection into tumor sites, offering precise delivery and localized treatment, which minimizes the impact on healthy tissues [1]. Injectable hydrogels, such as those containing gelatin–hydrazide (Gel-ADH) and aldehyde-functionalized polyethylene glycol (diBA-PEG), are widely used for their convenience and effectiveness in sustained drug release, particularly for chemotherapeutic agents like Doxorubicin [2].

In situ-forming hydrogels, like the glutathione–gellan gum conjugate (GSH-GG) with Doxorubicin, are engineered to gel at the target site within the body, providing a less invasive and targeted approach to treatment [4]. Deep subcutaneous injections of lanreotide Autogel demonstrate the method’s importance in delivering consistent, long-term therapy for conditions such as acromegaly and neuroendocrine tumors, highlighting the method’s widespread acceptance and usage [18,19,21,22,23,24].

For diseases like glioblastoma, hydrogels loaded with Carmustine are injected into the post-surgical resection cavity, ensuring direct and localized drug delivery to the affected area [7]. Similarly, the intracavity administration of Poloxamer 407 gel for nanoparticle-loaded drugs exemplifies the strategic approach to treating internal body cavities [8].

Ophthalmic drug delivery utilizes hydrogels for both topical application and intravitreal injections to address eye conditions effectively. Topical hydrogels containing drugs like metoprolol tartrate are applied directly to the eye, providing targeted treatment with minimal systemic exposure [30]. Intravitreal injections, such as those delivering bevacizumab within a pre-crosslinked hydrogel, offer a method for sustained release directly into the vitreous humor, treating retinal conditions effectively [33].

Pain management strategies employ hydrogels for both localized and systemic relief. Thermosensitive and bioadhesive gels containing lidocaine hydrochloride, for instance, are administered intraperitoneally to deliver targeted pain relief and anti-inflammatory effects within the peritoneal area [37]. Similarly, hydrogels with bupivacaine are injected intramuscularly or at specific nerve block sites to provide long-lasting pain control [39,40].

In contraceptive applications, subcutaneous injections of hydrogels loaded with levonorgestrel are utilized for their sustained-release properties, offering a long-term solution with minimal patient intervention [42,43]. This method’s efficacy and convenience underline its preference in clinical settings.

The diverse administration methods for long-acting gels in drug delivery highlight the sector’s innovative approach to enhancing therapeutic outcomes and patient experience. From precise tumor targeting to pain management and controlled contraceptive delivery, these methods illustrate the adaptability and effectiveness of hydrogel systems in addressing a broad spectrum of medical conditions.

## 5. Testing of the Long-Acting Gels

In vitro testing involves a variety of assessments, including drug release kinetics, gelation time, and pH-responsive behavior [2], with further studies exploring the near-infrared and pH-responsive release mechanisms alongside cytotoxic effects [3]. These tests also evaluate drug release under varying conditions [4], comparing the in vitro drug release and skin penetration against existing formulations [65], and delve into the drug encapsulation efficiency and cellular impact on cancer cells [66]. Degradation studies and sustained release evaluations are also prominent [12,13].

Cellular studies focus on biocompatibility and cytotoxicity [2], analyzing the effects on cancer cell inhibition, the immune response, and T cell activity [9]. Research extends to the effects on human melanoma cell growth and biocompatibility [6], with in vitro and in vivo examinations of protein release and tumor growth inhibition [10].

In vivo tests investigate cancer and tumor responses, evaluating slow-release formulations and their impact on tumor size [1], anticancer effects [3,4], tumor immune environment changes [8], and drug delivery kinetics [7]. These studies also cover the effectiveness of symptom relief and tumor progression in specific neuroendocrine tumors [18,19].

Diabetes and glucose control studies in vivo assess hypoglycemic effects, glycemic regulation [11], and the duration of drug release [12], focusing on blood glucose stability, weight management, and tissue analysis [13]. Pharmacokinetic and efficacy studies characterize sustained release and therapeutic effects [14,15,16,17].

Physicochemical and mechanical testing examines properties such as gelation, viscosity, and injectability [3,4,5], while clinical studies compare the long-term efficacy, safety, and symptom management of the developed therapies against existing therapies [18,19,20,21,22].

Hormonal and clinical efficacy studies monitor growth hormone and insulin-like growth factor levels, comparing different treatments’ effects on acromegaly symptoms and patients’ quality of life [23,24,25,26]. Insulin and glucose metabolism are assessed through resistance and beta-cell function tests [25].

Pharmacokinetic and drug release research involves studying the release rate and half-life of formulations [27], comparing these with other formulations, analyzing in vitro and in vivo kinetics [42,52], and assessing transdermal delivery and bioavailability [59,63].

In vivo and clinical efficacy studies evaluate analgesic effects, ovarian cycle suppression [39,43], and glucose control in infants [51], alongside hematocrit and testosterone level assessments [71].

Material and physicochemical characterization involves particle size, rheological property, and biochemical analyses [54,55,56,57,58,64,72], focusing on gelation, drug interaction, and controlled release [56,57,58,59,60].

User experience studies compare preferences for injection systems among different medications [28], while epidemiological research includes surveys and discussions on long-acting reversible contraceptive availability [45], sensory perceptions [69], and the acceptability and characteristics of products [70].

## 6. Therapeutic Outcomes

### 6.1. Enhanced Localized Treatment and Controlled Drug Release

Long-acting gels, such as Tam-Gel, BCNU-loaded hydrogels, Gel-ADH/diBA-PEG/LAP@DOX, and Lira-loaded hydrogels, have concentrated therapeutic effects in targeted areas, reducing systemic exposure and side effects while providing pH-dependent and sustained drug release. This approach is crucial for maintaining the therapeutic window and improving patient compliance [1,2,7,11].

### 6.2. Reduced Systemic Toxicity and Innovative Treatment Modalities

Studies have shown minimized systemic toxicity through localized and controlled drug release, enhancing treatment safety. The integration of nanotechnology with hydrogels has introduced novel therapies, including chemophotothermal therapy and immunotherapy, offering new possibilities for complex disease management like cancer [2,3,5,8,9].

### 6.3. Sustained and Extended Drug Release

Long-acting gels, such as EXT phospholipid gel, Ex4-C16 in DA-GC hydrogels, and other formulations, have demonstrated prolonged drug release, reduced frequent dosing needs and improving patient compliance. This extended release has been beneficial in various medical fields, from cancer to ophthalmology and pain management [14,15,30,31,37].

### 6.4. Localized and Targeted Therapy

These gels facilitate localized delivery, minimizing systemic effects and side effects, and enhancing treatment efficacy. Notable applications include lanreotide Autogel for neuroendocrine tumors and gels for targeted intraocular pressure control in ophthalmology [18,19,30,33].

### 6.5. Innovative Administration Routes and Diverse Therapeutic Applications

Oral and inhaled formulations, like oral insulin hydrogel microparticles and inhaled Ex4-C16-loaded DOCA-GC nanogels, offer non-invasive alternatives, improving the patient experience. These gels have been applied across a range of conditions, demonstrating versatility in treating chronic diseases such as diabetes, cancer, and glaucoma [14,16,17,18,30].

### 6.6. Extended Therapeutic Effects and Diverse Clinical Applications

Long-acting gels maintain sustained drug release over extended periods, which is essential for continuous treatment in conditions like HIV/AIDS and Parkinson’s disease. Their adaptability is evidenced in applications ranging from contraception in livestock to the treatment of schizophrenia and hypogonadism [42,43,47,52,53,71].

### 6.7. Improved Patient Compliance, Enhanced Wound Healing, and Antimicrobial Activity

The extended-release properties of these gels improve patient adherence, as seen in HIV/AIDS management. Additionally, technologies like GKNG and QAS-incorporated micro/nanofibers, along with AgNPs@CS/SCS hydrogels, have enhanced wound healing and shown broad-spectrum antibacterial activity, reducing the risk of infection in various treatments [48,49,54,55,64].

### 6.8. Sustained Drug Release

Systems including CNC/DPZ hydrogels and silk fibroin xerogels have demonstrated sustained drug release, indicating long-term therapeutic efficacy in treating chronic diseases [56,58].

## 7. Limitations

Hydrogel development faces scalability and manufacturing challenges, affecting their consistency and viability for clinical and commercial use. Long-term stability, efficacy, and safety data are insufficient, with ongoing research needed to confirm their long-term benefits. These systems undergo rigorous regulatory scrutiny, increasing development costs and delaying market entry. The mechanisms of drug release and action remain unclear, leading to variable patient responses and complicating treatment optimization. Notably, specific formulations like long-acting carteolol and timolol gel have raised safety concerns, underlining the need for comprehensive safety assessments. [20,25,29,32]. Additionally, issues with accessibility, distribution, and acceptance, especially in reproductive health, are influenced by cultural and logistical barriers [45,69,70].

## 8. Future Directions

Future research aims to develop hydrogel systems with improved targeting and personalization, enhancing specificity and reducing side effects. The focus is on creating biodegradable, safe hydrogels that minimize long-term adverse effects while improving patient outcomes. Exploring combination therapies and fostering interdisciplinary collaboration can advance hydrogel technology, particularly in complex diseases like cancer. Addressing regulatory and ethical considerations is crucial to ensuring equitable access to these therapies. Efforts should prioritize patient-centric development and extensive studies to better understand these therapies’ potential and limitations, facilitating their transition to mainstream clinical use [3,8].

## 9. Conclusions

Localized treatment and controlled drug release, as well as achievements in hydrogel technology, have transformed therapeutic approaches, enhancing efficacy while minimizing systemic exposure and side effects. From extended drug release to innovative administration routes, hydrogels have showcased immense potential across diverse clinical applications, promising improved patient compliance and therapeutic outcomes. However, scalability challenges and regulatory hurdles emphasize the need for continuous refinement and vigilance. Looking ahead, future directions emphasize the development of smarter, biodegradable hydrogel systems tailored to individual patient needs, alongside the exploration of combination therapies and interdisciplinary innovation. Addressing regulatory, ethical, and access considerations while prioritizing patient-centric development will be pivotal in realizing the full potential of hydrogel-based therapies, ensuring safer, more effective treatments for diverse medical conditions.

## Figures and Tables

**Table 1:** The study objective, gel compositions, and their method of administration in the treatment of cancer.

Objective	Gel Composition	Method of Administration	Ref.
To improve the antitumor effect of tamoxifen and reduce side effects in endocrine regulation.	Temperature-sensitive phase-change hydrogel of tamoxifen (Tam-Gel).	Intratumoral injection of Tam-Gel	[1]
To achieve controlled, long-term drug release for local cancer treatment with minimal side effects.	Gelatin–hydrazide (Gel-ADH), aldehyde-functionalized PEG (diBA-PEG), Laponite (LAP), and Doxorubicin (DOX).	Injectable hydrogel	[2]
To develop a long-term, controlled release system for chemophotothermal synergistic cancer therapy.	N-isopropylacrylamide (NIPAm), methacrylated poly-beta-cyclodextrin (MPCD), gold nanorods (GNRs), and adamantane-modified doxorubicin (AD-DOX).	Injectable, NIR/pH-responsive hydrogel	[3]
To provide long-acting cancer treatment with selective cytotoxicity towards cancer cells.	Glutathione-gellan gum conjugate (GSH-GG) with Doxorubicin hydrochloride (DOX).	In situ hydrogel	[4]
To enhance the aqueous solubility of melphalan and reduce its haemolytic activity for breast cancer treatment.	Chitosan (CS), glycerophosphate disodium salt (GP), and PEGylated melphalan conjugates (MLPEG 2000 and MLPEG 5000).	Injectable hydrogel	[5]
Enhance treatment of uveal melanoma by improving therapeutic delivery, reducing injection frequency, and achieving long-term inhibition of tumor progression.	In situ gelling hydrogel system composed of collagen and hyaluronic acid with curcumin-loaded nanoparticles.	In situ gelling hydrogel composite	[6]
Inhibiting glioblastoma recurrence post-resection.	Carmustine (BCNU) and BCNU-poly(lactic-co-glycolic acid) nanoparticles in pigskin extracellular matrix hydrogel.	Injection into resection cavity of glioblastoma	[7]
Suppressing postoperative tumor recurrence and metastasis via immunogenic chemotherapy.	Curcumin/doxorubicin-loaded nanoparticles (NanoCD) in Poloxamer 407 gel (NanoCD@Gel).	Intracavity administration of NanoCD@Gel	[8]
Provide a multi-effective, long-acting immunotherapy that improves immune function and inhibits cancer cell proliferation, migration, and recurrence.	Gold nanorods (AuNRs) with macrophage migration inhibitory factor (MIF) inhibitors in a sustained release hydrogel.	Single hydrogel-based injection	[9]
Develop a local protein delivery system to reduce systemic toxicity in cancer immunotherapies.	Redox-active, injectable gel (RIG) formed by a polyion complex of cationic polyamine-poly(ethylene glycol)-polyamine, anionic poly(acrylic acid), and a protein.	Injectable gel (RIG)	[10]
Improve controlled topical delivery of mupirocin with prolonged action and enhanced skin penetration.	Dammar gum–ethyl cellulose composite microsponge loaded gel formulation (D-MSPG) containing mupirocin.	Topical application of microsponge-based gel	[65]
Developing oral long-acting gels for colorectal cancer treatment.	Resveratrol–Soy protein wet granules encapsulated in sodium alginate dry films.	Oral in situ gelling films	[66]

**Table 2:** The study objective, gel compositions, and their method of administration for diabetes management.

Objective	Gel Composition	Method of Administration	Ref.
Constructing a long-acting antidiabetic system.	Poly(epsilon-caprolactone-co-glycolic acid)-poly(ethylene glycol)-poly(epsilon-caprolactone-co-glycolic acid) triblock copolymer hydrogel containing liraglutide.	Injectable thermosensitive hydrogel	[11]
Developing a long-acting injectable formulation for type II diabetes treatment.	Poly(lactic acid-co-glycolic acid)-poly(ethylene glycol)-poly(lactic acid-co-glycolic acid) triblock copolymer hydrogel with exenatide and zinc acetate.	Injectable block copolymer hydrogel	[12]
Creating a long-acting delivery system for diabetes treatment with high drug bioactivity.	PLGA-PEG-PLGA and PCLA-PEG-PCLA mixed hydrogels with exenatide-loaded microspheres.	Depot-gel-in-Ms-in-Matrix-gel system injection	[13]
Developing a sustained-release exenatide formulation for long-term diabetes management.	Phospholipid S100, medium chain triglyceride (MCT), and 85% ethanol gel containing exenatide.	Subcutaneous injection	[14]
Creating a long-acting anti-diabetic system with sustained exendin-4 release.	Decanoic acid-modified glycol chitosan hydrogels with palmityl-acylated exendin-4 (Ex4-C16).	Subcutaneous injection	[15]
Developing a long-acting inhalable anti-diabetic system with exendin-4.	Deoxycholic acid-modified glycol chitosan nanogels containing palmityl acylated exendin-4 (Ex4-C16).	Pulmonary administration	[16]
Enhancing oral insulin delivery efficiency in diabetes treatment.	Polysaccharide hydrogel microparticles with physical and chemical crosslinking containing insulin.	Oral administration of hydrogel microparticles	[17]

**Table 3:** The study objective, gel compositions, and their method of administration for the treatment of neuroendocrine and other tumors.

Objective	Gel Composition	Method of Administration	Ref.
Assessing the efficacy and safety of lanreotide Autogel in treating neuroendocrine tumors.	Lanreotide Autogel.	Deep subcutaneous injection	[18]
Evaluating efficacy, safety, and tolerability of lanreotide Autogel in metastatic neuroendocrine tumor (NET) patients.	Lanreotide Autogel.	Deep subcutaneous injection	[19]
Analyzing biochemical efficacy of lanreotide depot/Autogel in acromegalic patients naive to somatostatin-receptor ligands.	Lanreotide depot/Autogel.	Subcutaneous injection	[20]
Assessing the efficacy and safety of lanreotide Autogel in managing acromegaly.	Lanreotide Autogel.	Deep subcutaneous injection	[21]
Four-year follow-up of acromegalic patients treated with lanreotide Autogel.	Lanreotide Autogel.	Deep subcutaneous injection	[22]
Evaluating lanreotide Autogel in somatostatin analogue-naive acromegaly patients.	Lanreotide Autogel.	Deep subcutaneous injection every 4–8 weeks	[23]
Comparing efficacy and tolerability of lanreotide Autogel with octreotide LAR in acromegaly treatment.	Lanreotide Autogel.	Deep subcutaneous injection	[24]
Investigating the effects of lanreotide Autogel on glucose tolerance and insulin resistance in acromegaly.	Lanreotide Autogel.	Not specified	[25]
Evaluating the efficacy of lanreotide Autogel and octreotide LAR in controlling GH secretion in acromegaly.	Lanreotide Autogel.	Not specified	[26]
Developing a long-acting subcutaneous octreotide delivery system to improve patient compliance and reduce injection pain.	Tetra-PEG hydrogel microspheres covalently attached to octreotide.	Subcutaneous injection	[27]
Assessing nurse preferences between the new lanreotide Autogel syringe and octreotide LAR syringe in a simulated-use study.	Not applicable (study focuses on syringe design, not gel composition).	Not applicable (survey study)	[28]
Comparing lanreotide Autogel and octreotide LAR for acromegaly treatment.	Lanreotide Autogel.	Subcutaneous injection	[29]

**Table 4:** The study objective, gel compositions, and their method of administration for ophthalmology and vision health.

Objective	Gel Composition	Method of Administration	Ref.
Developing long-acting ophthalmic gels for extended drug contact and slow release.	Metoprolol tartrate with Carbopol 934 and Pluronic F127.	Ophthalmic application	[30]
Formulating a long-acting ophthalmic gel of atenolol to improve drug concentration at the action site.	Atenolol with carboxymethylcellulose and sodium alginate.	Ophthalmic application	[31]
Assessing the impact of carteolol long-acting and timolol gel-forming solutions on ocular aberrations.	Not specified (study focuses on comparing two formulations).	Ophthalmic application	[32]
Developing an intravitreal injectable hydrogel rod for long-acting delivery of bevacizumab to the retina.	Pre-crosslinked hydrogel rod containing bevacizumab.	Intravitreal injection	[33]
Developing long-acting intravitreal treatments to reduce frequent administration in ocular diseases.	BAY224 encapsulated in biodegradable silica microparticles within a silica–silica hydrogel composite.	Intravitreal injection	[34]
Creating a long-acting subconjunctival in situ gelling implant for glaucoma treatment.	Bimatoprost-loaded nanovesicular thermosensitive in situ gelling implant.	Subconjunctival injection	[35]
Improving topical treatments for dry eye disease with a long-acting mucoadhesive thermogel.	Gelatin, poly(N-isopropylacrylamide), and lectin Helix pomatia agglutinin-based mucoadhesive drug delivery system.	Topical administration	[36]

**Table 5:** The study objective, gel compositions, and their method of administration for pain management and anesthesia.

Objective	Gel Composition	Method of Administration	Ref.
Developing a long-acting gel for postoperative pain management in abdominal surgeries.	Lidocaine hydrochloride and dexamethasone sodium phosphate in a thermosensitive and bioadhesive gel consisting of poloxamer 407, hydroxypropyl methylcellulose, and polyvinyl pyrrolidone.	Intraperitoneal injection	[37]
Formulating a thermogel for long-acting analgesia with levobupivacaine.	Levobupivacaine in a thermosensitive gel composed of poloxamer.	Not specified	[38]
Achieving long-acting local analgesia.	Intelligent hydrogel with bupivacaine microcrystals and calcium carbonate as a pH regulator.	Intramuscular injection	[39]
Developing precision-guided long-acting analgesia.	Gel-immobilized bupivacaine-loaded microspheres within PLGA-PEG-PLGA gel.	Injection at the sciatic nerve block site	[40]
Creating a composite for sustained analgesia.	Hydrogel/microsphere composite co-encapsulating bupivacaine and dexmedetomidine.	Not specified	[41]
Managing chronic wound pain with topical gel.	Lidocaine in a TRI-726 matrix gel.	Topical application	[67]
Determining pharmacokinetics of butorphanol in birds.	Butorphanol tartrate in poloxamer 407 gel.	Subcutaneous injection	[68]

**Table 6:** The study objective, gel compositions, and their method of administration for contraception, sexual health, and HIV prevention.

Objective	Gel Composition	Method of Administration	Ref.
Developing long-term injectable contraception for animals.	PLGA-PEG-PLGA triblock copolymer loaded with levonorgestrel (LNG).	Subcutaneous injection	[42]
Creating an effective contraceptive option for cotton-top tamarins.	Injectable gel matrix with levonorgestrel, composed of polylactic-co-glycolic acid, triethyl citrate, and N-methylpyrrolidone.	Subcutaneous injection	[43]
Assessing long-acting reversible contraceptive (LARC) utilization among Ethiopian women.	Not applicable (study focused on utilization, not on gel composition).	Not applicable (survey study)	[44]
Developing long-acting bioadhesive vaginal gels for overactive bladder.	Oxybutynin in a mucoadhesive gel composed of chitosan, hydroxypropyl methylcellulose (HPMC K100 M), and Poloxamer 407.	Vaginal application	[46]
Understanding user experience of vaginal gels and intravaginal rings.	Not specified (study focuses on user perceptions, not gel composition).	Subcutaneous injection	[47]
Opinions of long-acting topical vaginal gels.	Not specified (study focuses on perceptions, not gel composition).	Subcutaneous injection	[48]
Long-acting injectable depot system for anti-HIV drug.	Tenofovir alafenamide–chitosan nanoparticle-loaded oleogels with sesame oil and ethyl cellulose.	Intradermal injection	[50]

**Table 7:** The study objective, gel compositions, and their method of administration for other therapeutic areas.

Objective	Gel Composition	Method of Administration	Ref.
Treating severe neonatal hyperinsulinism with long-acting lanreotide.	Long-acting lanreotide Autogel (LAN-ATG).	Subcutaneous injection	[51]
Developing a long-acting injectable gel of rasagiline for Parkinson’s disease.	Rasagiline in situ gel with aluminum hydroxide for sustained release.	Subcutaneous injection	[52]
Analyzing long-acting risperidone formulation for schizophrenia.	Subcutaneous formulation of risperidone (RBP-7000) in Atrigel.	Subcutaneous injection	[53]
Development of a long-acting wound healing gel	Ginkgo biloba/Sodium Alginate Nanocomplex Gel	Topical application	[54]
Creation of a long-acting, broad-spectrum antimicrobial wound dressing	Poly (epsilon-caprolactone)/gelatin micro/nanofibers with Trimethoxysilylpropyl octadecyldimethyl ammonium chloride	Topical application	[55]
Sustained drug delivery through a pH-controlled gel structure	Cellulose nanocrystal gel integrated with donepezil hydrochloride	Subcutaneous injection	[56]
Long-acting alginate in situ forming gel injectable with modulated gelation time	Alginate gel with glucono-d-lactone, pyridoxal 5‘-phosphate, and paliperidone palmitate	Injectable (subcutaneous)	[57]
Develop a long-acting estradiol delivery system for therapeutic use.	Silk fibroin xerogels with ethanol for gelation and estradiol incorporation.	Not specified	[58]
Enhance bioavailability and prolong the action of nifedipine.	Nifedipine-loaded gelatin microcapsule, coated with Eudragit acrylic resin.	Oral	[59]
Improve steroid delivery to the paranasal sinuses for chronic sinusitis treatment.	Thermogel with mometasone furoate embedded in poly(lactic-co-glycolic acid) microspheres within a poly(N-isopropylacrylamide)-based hydrogel.	Injectable (local)	[60]
Long-acting delivery of moxifloxacin to avoid frequent dosing.	Moxifloxacin in a long-acting poloxamer 407 gel formulation.	Injectable (subcutaneous)	[61]
Create a long-acting, slow-release material for chlorine dioxide.	Carboxymethylcellulose–agar hydrogel with chlorine dioxide in bagasse pulp-based superabsorbent resin.	Not specified	[62]
Enable transdermal long-acting delivery of a hydrophobic drug.	Hydrogel-forming microarray patches with solid dispersion reservoirs for atorvastatin delivery.	Transdermal	[63]
Development of a long-acting, multifunctional antibacterial agent	Chitosan nanogel with in situ generated silver nanoparticles (AgNPs)	Not specified	[64]
Comparing testosterone therapies on hematopoiesis in hypogonadal men.	Not applicable (study compares two testosterone therapy forms, not gel composition).	Transdermal application and intramuscular injection	[71]
Long-acting delivery to mucosal tissues with bioadhesive eutectogels	Eutectogels containing gelatin, glycerine, choline hydrochloride–glycerol DES, and curcumin nanocrystals	Mucosal application	[72]
